# Demonstration of Impact Through the Evaluation of the 2020-2021 Africa Cancer Research and Control ECHO

**DOI:** 10.1200/GO.22.43000

**Published:** 2022-05-05

**Authors:** Nwamaka Lasebikan, Annet Nakaganda, Temidayo Fadelu, Paulette Ibeka, Laura Haskins, Mishka Cira

**Affiliations:** ^1^ University of Nigeria Teaching Hospital Enugu/ African Cancer Research and Control ECHO, Enugu, Nigeria; ^2^ Uganda Cancer Institute, Kampala, Uganda; ^3^ Dana-Farber/Harvard Cancer Center, Boston, MA; ^4^ Clinton Health Access Initiative, Abuja, Nigeria; ^5^ Johns Hopkins School of Nursing, Baltimore, MD; ^6^ US National Cancer Institute, Rockville, MD

## Abstract

The Africa Cancer Research and Control ECHO is a virtual community of practice and knowledge dissemination platform for cancer professionals. Each year, the steering committee completes an evaluation to elicit feedback and inform continuous program improvement. The 2020-2021 evaluation included for the first time a qualitative component, allowing documentation of concrete utilization of knowledge gained from the Africa Cancer ECHO to advance cancer control efforts.

**METHODS:**

Participants were invited to complete a survey about satisfaction, knowledge acquisition, knowledge application, and outcomes of the Africa Cancer ECHO. In addition, six steering committee members completed semi-structured interviews with a focus on program outcomes.

**RESULTS:**

A total of 226 unique people from over 40 organizations participated in the Africa Cancer ECHO during 2020-2021, with 19 sessions hosted on topics across the cancer control continuum. Twenty-two participants representing nine countries and multiple sectors of cancer research and control responded to the survey. They reported participating to gain knowledge (57%), network (21%) and strongly agreed that sessions were relevant and engaging (100%). Reported limitations included obstacles of time (74%), technology (13%) and limited opportunities to engage with partners and participants (50%). Participants applied the knowledge gained in various ways including: adding survivorship to their National Cancer Control Plans (for the first time); adapting training materials to initiate a patient navigation program, partnering to strengthen brachytherapy capacity at the national level and collaboratively conducting a situational analysis of cancer survivorship in the Africa region.

**CONCLUSION:**

The Africa Cancer ECHO provides a relevant and engaging platform which encourages community and the exchange of socio-culturally adapted best practices in cancer research and control in the African region. The mixed methods evaluation approach has deepened understanding of how knowledge exchanged in the ECHO sessions is utilized and applied. More targeted recruitment and retention, as well as facilitation of collaborations beyond the sessions will further strengthen this community.

